# Knowledge, attitudes and practices of the Chinese public with respect to coronavirus disease (COVID-19): an online cross-sectional survey

**DOI:** 10.1186/s12889-020-09961-2

**Published:** 2020-11-30

**Authors:** Huiming Gao, Rujun Hu, Ling Yin, Xiaoli Yuan, Hao Tang, Lan Luo, Mei Chen, Di Huang, Ying Wang, Anyong Yu, Zhixia Jiang

**Affiliations:** 1 Department of Nursing, Affiliated Hospital of Zunyi Medical University, Zunyi, 563000 Guizhou China; 2 Department of Emergency, Affiliated Hospital of Zunyi Medical University, Zunyi, 563000 Guizhou China

**Keywords:** COVID-19, Knowledge, Attitude, Practice, Survey

## Abstract

**Background:**

Coronavirus disease (COVID-19) has become a pandemic. The knowledge, attitudes, and practices (KAP) of the public play a major role in the prevention and control of infectious diseases. The objective of the present study was to evaluate the KAP of the Chinese public and to assess potential influencing factors related to practices.

**Methods:**

A cross-sectional online survey was conducted in China in February 2020 via a self-designed questionnaire comprising 33 questions assessing KAP.

**Results:**

For the 2136 respondents from 30 provinces or municipalities in China, the accurate response rate for the knowledge section ranged from 72.7 to 99.5%, and the average was 91.2%. Regarding attitude section, the percentage of positive attitudes (“strongly agree” and “agree”) ranged from 94.7 to 99.7%, and the average value was 98.0%. The good practices (“always” and “often”) results ranged from 76.1 to 99.5%, and the average value was 96.8%. The independent samples t-test revealed that gender and ethnic differences had no effect on knowledge, attitude or behaviour (*P* > 0.05). However, knowledge was associated with age (t = 4.842, *p* < 0.001), marital status (t = − 5.323, *p* < 0.001), education level (t = 8.441, *p* < 0.001), occupation (t = − 10.858, *p* < 0.001), and place of residence (t = 7.929, *p* < 0.001). Similarly, attitude was associated with marital status (t = − 2.383, *p* = 0.017), education level (t = 2.106, *p* = 0.035), occupation (t = − 4.834, *p* < 0.001), and place of residence (t = 4.242, *p* < 0.001). The multiple linear regression analysis results showed that the factors influencing practices were knowledge (t = − 3.281, *p* = 0.001), attitude (t = 18.756, *p* < 0.001), occupation (t = − 3.860, *p* < 0.001), education level (t = 3.136, *p* = 0.002), and place of residence (t = 3.257, *p* = 0.001).

**Conclusions:**

The Chinese public exhibited a good level of knowledge of COVID-19, a positive attitude, and high adherence to good practices. COVID-19-related knowledge, attitudes and practices were affected by age, marital status, education level, occupation, and place of residence to varying degrees. In addition, practices were affected by knowledge and attitudes towards COVID-19.

**Supplementary Information:**

The online version contains supplementary material available at 10.1186/s12889-020-09961-2.

## Introduction

Since December 2019, coronavirus disease (COVID-19) has become a pandemic, and patients with confirmed disease and those that are infected but asymptomatic are the main sources of infection [1]. The routes of transmission are diverse and include close contact, droplets from asymptomatic or oligosymptomatic patients and possibly aerosols in health care environments [1], and the population is generally susceptible to infection. According to WHO statistics, as of 23 March 2020, the number of confirmed COVID-19 cases had reached 332,930 worldwide [2]. Among these cases, 81,601 were in China, and the remaining 251,329 cases were from more than 180 different countries, including Italy, the United States of America, Spain, Germany, and Iran. The disease has resulted in 14,510 deaths so far, and the fatality rate is approximately 4.35% [2]. Obviously, COVID-19 has become a global public health issue.

Because the population is generally susceptible to COVID-19, it is extremely challenging to prevent and control the spread of this infectious disease. Currently, no vaccine has been developed for COVID-19, and no antiviral drug is specifically recommended. Therefore, applying preventive measures, such as avoiding contact with people with confirmed or suspected infection, practising good hand hygiene, observing respiratory etiquette, and cleaning and disinfecting surfaces, is of utmost importance to reduce the spread of the disease [3, 4]. The existing literature has indicated that knowledge, attitude and risk perception with respect to infectious diseases are significantly correlated with protective behaviour [5–7], and the behaviours of the public and people in potential risk groups can play a major role in both the prevention and control of infectious diseases [8]. Therefore, it is important to assess the public’s knowledge, attitudes, and practices (KAP) regarding COVID-19. However, little is known about the KAP among the Chinese public regarding COVID-19. Specifically, the present study was performed to evaluate KAP among the Chinese public and to assess potential influencing factors related to preventive behaviours. The results of the study can provide information about the effectiveness of the health education efforts of health authorities in China and about how to design evidence-based interventions to reduce the risk of contracting COVID-19.

## Methods

### Study design and participants

This study utilized a cross-sectional online survey conducted on a special questionnaire survey platform (Wenjuanxing, https://www.wjx.cn/) in China. Convenience sampling was used to select potential respondents. The inclusion criteria were ① ≥18 years old, ② able to use a smartphone, and ③ able to understand the content of the questionnaire. The exclusion criteria were ① unable to fill in the questionnaire due to illness or other reasons and ② unwilling to participate in the research. According to the Kendall sample estimation method for multivariate analysis, the minimum sample size was required to be 10 times the number of variables [9]. The questionnaire applied in the study included 33 variables, and the minimum sample size for this survey was therefore 330.

### Instrument

Based on the research purpose, the researchers designed the questionnaire themselves (see Additional File 1). The survey consisted of four sections: demographic variables and knowledge, attitudes and practices with respect to COVID-19. The demographic variables included sex, age, ethnicity, marital status, education level, occupation and place of residence. There were 13 items in the knowledge section, and each item contained 3 options, namely, “true”, “false” and “don’t know”; 1 point was given for a correct answer, and 0 points were awarded for an incorrect answer or a “don’t know” response. The total score of this section ranged from 0 to 13, and higher scores were correlated with more knowledge. The attitude section included five items, and a Likert scale was used to assess the level of agreement with the statements; response options ranged from 1 (strongly disagree) to 5 (strongly agree). The total scores in this section could range from 5 to 25, and higher scores indicated a more positive attitude. The practices section included 15 items, and a Likert scale was used to assess the level of agreement with the statements; the response options ranged from 1 (never) to 4 (always). The total scores in this section could range from 15 to 60, with higher scores correlating with better protective actions being taken. After the questionnaire draft was designed, a total of 20 public individuals with different sociodemographic backgrounds were selected to answer the questionnaire by convenience sampling to test its intelligibility, generalizability and time needed for completion. The content validity index (CVI) of the questionnaire was confirmed by 9 experts on our research team. Before the formal survey, we conducted a pilot study including 50 people to evaluate the internal consistency reliability (Cronbach’s α). Finally, the CVI was 0.94, and the Cronbach’s α value was 0.846.

### Data collection

The self-designed questionnaire was input into an online questionnaire survey platform (Wenjuanxing, https://www.wjx.cn/), and the questionnaires were sent to potential respondents through WeChat by convenience sampling in February 2020. Online informed consent was presented to the respondents before they completed the questionnaire.

### Statistical analysis

SPSS version 22.0 (IBM Corp., Armonk, NY, USA) was used for all statistical analyses. The categorical data are described by frequencies and composition ratios. The continuous data are described as the means and standard deviations. The t-test was used to analyse KAP differences in regards to COVID-19 among groups with different demographic characteristics. Multiple linear regression analysis was used to explore the factors influencing the public’s COVID-19 prevention practices. All statistical tests were two-sided, with *p* ≤ 0.05 indicating statistical significance.

## Results

### Participant sociodemographic information

A total of 2136 survey responses from 30 provinces or municipalities in China were received. The top five participating provinces and municipalities were Guizhou (1701, 79.6%), Chongqing (165, 7.7%), Sichuan (130, 6.1%), Hubei (30, 1.4%), and Henan (25, 1.2%). The sociodemographic details of the 2136 participants are summarized in Table 1.

**Table 1:** Sociodemographic information of the participants (*N* = 2136)

Variable	n (%) or Mean ± SD
**Sex**	
Male	467 (21.9)
Female	1669 (78.1)
**Age (year)**	33.1 ± 8.8
**Ethnicity**	
Han	1830 (85.7)
Other	306 (14.3)
**Marital status**	
Married	1473 (69.0)
Unmarried	593 (27.8)
Divorced	65 (3.0)
Widowed	5 (0.2)
**Education level**	
Middle school and below	94 (4.4)
High school/technical secondary school	113 (5.3)
Junior college	333 (15.6)
Bachelor’s degree	1259 (58.9)
Master’s degree and above	337 (15.8)
**Occupation**	
Medical staff	1228 (57.5)
Worker	34 (1.6)
Farmer	23 (1.1)
Self-employed	120 (5.6)
Student	176 (8.2)
Employee of an enterprise or institution	393 (18.4)
Unemployed or retired	33 (1.5)
Other	129 (6.0)
**Place of residence**	
City	1740 (81.5)
Countryside or town	396 (18.5)

*SD* standard deviation

### Knowledge of the public regarding COVID-19

The accurate response rate for the knowledge section ranged from 72.7% (*family gatherings may spread infection*) to 99.5% (*most people infected present with fever, fatigue, and dry cough as the main symptoms*), and the average accuracy rate was 91.2%. The accurate response rate was greater than 80% for all items except for “family gatherings may spread infection” and “a suspected case can be ruled only out after two consecutive negative tests of respiratory pathogenic nucleic acid (at least one day apart)” as shown in Table 2.

**Table 2:** Knowledge level of respondents regarding COVID-19 (*N* = 2136)

Items	Accuracy n (%)
1. The source of infection is primarily confirmed and asymptomatic patients	1919 (89.8)
2.Transmission mainly occurs through respiratory droplets and close contact	2108 (98.7)
3. The population is generally susceptible to infection	2001 (93.7)
4. It is highly infectious and spreads quickly	2121 (99.3)
5. Washing hands frequently, wearing masks and other measures can effectively prevent infection	2115 (99.0)
6. Family gatherings may spread infection	1553 (72.7)
7. The incubation time of the disease is 1–14 days, typically 3–7 days	2056 (96.3)
8. Most people infected present with fever, fatigue, and dry cough as the main symptoms	2125 (99.5)
9. Most patients have a good prognosis, while a few are in critical condition	1875 (87.8)
10. A suspected case can be ruled only out after two consecutive negative tests of respiratory pathogenic nucleic acid (at least 1 day apart)	1659 (77.7)
11. Suspected and confirmed patients should be isolated and treated in designated hospitals with effective isolation and protective conditions	2111 (98.8)
12. If you develop a fever during the outbreak, you can take your own medicine	1779 (83.3)
13. Medical alcohol at a concentration of 75% can effectively inactivate the virus	1911 (89.5)
Average accuracy	91.2

### Attitude of the public towards COVID-19

The proportion of positive attitudes (strongly agree and agree) ranged from 94.7% (*It is believed that the outbreak will soon be contained*) to 99.7% (*I am willing to cooperate with the relevant departments to take prevention and control measures*), and the average value was 98.0%, as shown in Table 3.

**Table 3:** The public’s attitude towards COVID-19 (*N* = 2136)

Items	Strongly agree n(%)	Agree n(%)	Not sure n(%)	Disagree n(%)	Strongly disagree n (%)
1. I pay close attention to the development of the epidemic situation	1995 (93.4)	134 (6.3)	4 (0.2)	0 (0)	3 (0.1)
2. I think I am playing an important role in controlling the epidemic	1754 (82.1)	340 (15.9)	38 (1.8)	2 (0.1)	2 (0.1)
3. I fear infection for myself and my family	1809 (84.7)	273 (12.8)	41 (1.9)	7 (0.3)	6 (0.3)
4. It is believed that the outbreak will soon be contained	1699 (79.5)	325 (15.2)	96 (4.5)	11 (0.5)	5 (0.2)
5. I am willing to cooperate with the relevant departments to take prevention and control measures	1956 (91.6)	172 (8.1)	5 (0.2)	0 (0)	2 (0.1)
Average rate	86.3	11.7	1.7	0.2	0.2

### Practices of the public with respect to COVID-19

The proportion of good practices (always and often) ranged from 76.1% (*home environment disinfection*) to 99.5% (*wash hands*), and the average value was 96.8%. The good practice adherence rate of all items was more than 80% except for “monitor body temperature”, “appropriate exercise”, and “home environment disinfection”, as shown in Table 4.

**Table 4:** The public’s COVID-19 prevention practices (*N* = 2136)

Items	Always n (%)	Often n (%)	Sometimes n (%)	Never n (%)
1. After the outbreak, stay at home to prevent infection	1752 (82.0)	301 (14.1)	63 (2.9)	20 (0.9)
2. Wear a mask when going out	2072 (97.0)	51 (2.4)	9 (0.4)	4 (0.2)
3. Wash hands	1970 (92.2)	155 (7.3)	10 (0.5)	0 (0)
4. Seek medical advice when symptoms such as fever and cough appear	1870 (87.5)	161 (7.5)	90 (4.2)	15 (0.7)
5. Monitor body temperature	1273 (59.6)	406 (19.0)	353 (16.5)	104 (4.9)
6. Open windows to keep the air fresh	1823 (85.3)	280 (13.1)	32 (1.5)	0 (0)
7. Rest properly and don’t stay up late	1498 (70.1)	385 (18.0)	229 (10.7)	24 (1.1)
8. Appropriate exercise	1287 (60.3)	406 (19.0)	407 (19.1)	36 (1.7)
9. Home environment disinfection	1104 (51.7)	522 (24.4)	448 (21.0)	62 (2.9)
10. Reduce time in airtight, airless environments	1780 (83.3)	244 (11.4)	54 (2.5)	58 (2.7)
11. Reduce visits to crowded places	1831 (85.7)	218 (10.2)	43 (2.0)	44 (2.1)
12. Avoid direct contact with public facilities that may be infected, such as elevator buttons and stair railings	1673 (78.3)	323 (15.1)	107 (5.0)	33 (1.5)
13. Active quarantine after contact with high-risk groups	1923 (90.0)	143 (6.7)	44 (2.1)	26 (1.2)
14. Cover mouth and nose when coughing or sneezing	1944 (91.0)	162 (7.6)	20 (0.9)	10 (0.5)
15. Keep warm and avoid catching cold	1965 (92.0)	161 (7.5)	6 (0.3)	4 (0.2)
Average rate	87.4	9.4	2.1	1.1

### Single-factor analysis of the factors influencing knowledge, attitudes and practices

Independent samples t-test analysis indicated that gender and ethnic differences had no effect on KAP. The knowledge score of the < 32-year-old group was lower than that of the ≥32-year-old group, and the difference was statistically significant. Marital status, education level, occupation and place of residence all had impacts on the KAP scores, and the differences were statistically significant, as shown in Table 5.

**Table 5:** Univariate analysis of the factors influencing knowledge, attitudes and practices (*N* = 2136)

Items	Number	Statistical value	Knowledge (mean ± SD)	Attitudes (mean ± SD)	Practices (mean ± SD)
**Sex**					
Male	467		11.78 ± 1.30	24.16 ± 1.61	55.37 ± 5.24
Female	1669		11.88 ± 1.15	24.19 ± 1.54	55.86 ± 4.93
		t	−1.593	−0.406	−1.782
		P	0.112	0.685	0.075
**Age (year)**^a^					
<32	999		11.73 ± 1.22	24.21 ± 1.70	55.87 ± 5.23
≥ 32	1137		11.98 ± 1.34	24.16 ± 1.41	55.65 ± 4.80
		t	4.842	−0.700	−0.999
		P	< 0.001	0.484	0.318
**Ethnicity**					
Han	1830		11.87 ± 1.16	24.17 ± 1.53	55.75 ± 4.94
Other	306		11.77 ± 1.30	24.28 ± 1.70	55.76 ± 5.40
		t	1.365	−1.164	−0.036
		P	0.173	0.245	0.971
**Marital status**					
Married	1473		11.96 ± 1.10	24.23 ± 1.43	55.93 ± 4.75
Unmarried or divorced or widowed	663		11.64 ± 1.32	24.07 ± 1.79	55.35 ± 5.52
		t	−5.323	−2.383	−2.101
		P	< 0.001	0.017	0.036
**Education level**					
Below junior college	207		11.01 ± 1.56	23.97 ± 1.71	53.53 ± 6.32
Junior college and above	1929		11.95 ± 1.10	24.21 ± 1.53	55.99 ± 4.78
		t	8.441	2.106	6.803
		P	< 0.001	0.035	< 0.001
**Occupation**					
Medical staff	1228		12.11 ± 0.93	24.33 ± 1.32	56.53 ± 4.27
Other	908		11.53 ± 1.39	23.99 ± 1.80	54.70 ± 5.69
		t	−10.858	−4.834	−8.497
		P	< 0.001	< 0.001	< 0.001
**Place of residence**					
City	1740		11.98 ± 1.05	24.27 ± 1.38	56.14 ± 4.56
Countryside or town	396		11.34 ± 1.54	23.80 ± 2.13	54.03 ± 6.33
		t	7.929	4.242	6.272
		P	< 0.001	< 0.001	< 0.001

^a^The median age of the subjects was 32 years

### Multiple linear regression analysis of the factors influencing practice (*N* = 2136)

The statistically significant indicators of knowledge and attitudes from the results of the single-factor analysis were incorporated into the multifactor model. With the practices score as the dependent variable, marital status (married = 1, single/widowed/divorced = 2), education (below junior college = 1, college degree and above = 2), occupation (medical staff = 1, other professions = 2), place of residence (city = 1, countryside/town = 2), knowledge (continuous variable), and total attitude score (continuous variable) were the six independent variables. Multiple linear regression was conducted by the stepwise entry method with a selection level of 0.05 and an elimination level of 0.10. The results showed that the regression model was statistically significant (*F* = 104.141, *P* < 0.001, adjusted *R*^*2*^ = 0.195), and the five independent variables included in the model had statistically significant effects on practice (*P* < 0.05), as shown in Table 6.

**Table 6:** Multiple linear regression analysis of the factors influencing practice

Variable	Partial regression coefficient (B)	Standard error (SE)	Standardized partial regression coefficient (beta)	t	P
Constant	23.537	2.009	–	11.716	< 0.001
Attitude score	1.195	0.064	0.371	18.756	< 0.001
Occupation	−0.838	0.217	−0.083	−3.860	< 0.001
Knowledge score	0.286	0.087	0.068	3.281	0.001
Place of residence	−0.867	0.266	−0.067	−3.257	0.001
Education level	1.130	0.360	0.067	3.136	0.002

## Discussion

The current study was conducted to assess and compare the KAP scores of different population groups with respect to COVID-19 in China. This evaluation was essential to improve protective practices for preventing COVID-19 infection among the public because no vaccine is available and no specific treatment is being offered against the disease at present. In our study, 2136 people participated, and the response efficiency was 100%.

The results of this study revealed that the Chinese public has good knowledge of COVID-19, with an overall correct response rate of 91.2%. This result may be attributed to the demographic characteristics of the participants (90.3% junior college degree or higher, 57.5% medical staff, and 81.5% urban residents) because significant associations between these demographic variables and KAP towards COVID-19 were verified in the present study. This result coincides with those of other similar studies performed among public and healthcare professionals in China and abroad [10–14]. The result was expected because Chinese governmental offices at all levels have released relevant education materials in a timely manner and have delivered COVID-19-related content through various channels, including television, the internet, WeChat, and publicity boards, since the outbreak began. The accuracy rate of 11 items ranged between 83.3 and 99.5%, with two items falling below this range, “family gatherings may spread infection” and “a suspected case can be ruled only out after two consecutive negative tests of respiratory pathogenic nucleic acid (at least one day apart)”. On the one hand, this result implies that the public has incomplete knowledge of COVID-19, especially regarding some professional topics, and still needs further education. On the other hand, this infectious disease has caused infection within family groups [15, 16]. Hence, health authorities should further increase publicity to raise public awareness of the disease.

In the present study, participants showed extremely positive attitudes towards COVID-19. A total of 99.7% of the public paid close attention to the development of the epidemic situation, 98.0% thought they played an important role in controlling the epidemic, 94.7% believed that the outbreak would soon be contained, and 99.7% expressed willingness to cooperate with the relevant departments to take prevention and control measures. The results are similar to those of other published studies [10, 17–19], likely because the Chinese government at different levels has attached great importance to the epidemic and adopted strict prevention and control measures in a timely manner against the disease after the outbreak [20]. In addition, with the COVID-19 pandemic and media reports, the Chinese public understands the severity of the epidemic. Therefore, they desire to actively participate in epidemic prevention and control. In addition, 97.5% of the public expressed fear of infection for themselves and their families, indicating that health authorities should continue to organize corresponding health education and publicity to prevent fear of spreading.

The majority of the public adhered to good practices with respect to COVID-19 infection, potentially because they had good COVID-19 knowledge and a positive attitude, which ultimately translates into good practice. However, the least common practice among participants was “home environment disinfection”. Perhaps the shortage of protective equipment, such as medical alcohol and chlorine-containing disinfectants, during the outbreak made it difficult to disinfect the home environment. This result suggests that during infectious disease outbreaks, government departments should try their best to provide sufficient supplies of protective equipment so that the public can take protective actions.

Further analysis found that age was a factor influencing the public’s grasp of COVID-19. The population younger than 32 years old (the median age of the study subjects) had less knowledge than their counterparts, which is potentially attributed to the increases in social experience and knowledge reserves with age. Marital status, education, occupation, and place of residence all had impacts on KAP. Married people had a better grasp of knowledge, more active protective attitudes, and higher adherence rates to protective behaviours than unmarried, divorced, or widowed individuals. This may be because married people have the responsibility of caring for their families in addition to self-protection. Therefore, they tend to learn more about protection, have a more positive attitude, and engage in proactive protective actions. People with a college degree or above had better KAP than their counterparts. Higher degrees may correlate with broader knowledge and a stronger learning ability, making it easier to grasp the relevant knowledge regarding COVID-19, adopt a protective attitude and be more positive. It has been suggested that health education should be targeted at people with different educational levels and different needs for health education. For the less educated population, easy-to-understand publicity materials may be more effective [21]. Medical personnel had more knowledge, better attitudes, and higher behaviour scores than non-medical respondents because medical personnel generally have a college degree or higher and have received more professional medical training. Hence, in the process of preventing and controlling COVID-19, the average person needs more education on knowledge regarding COVID-19 than medical staff. Compared with rural or urban residents, those living in cities had higher KAP scores, which was potentially due to the following reasons. (a) Information sources are more available and spread faster in cities, and people can obtain first-hand information quickly [22]. (b) The cultural literacy of city residents is generally higher than that of people living in rural or urban areas [23]. (c) The composition of people in the city is more complex, and the population density is high, which increases the likelihood of COVID-19 dissemination. As a result, citizens are more proactive in epidemic prevention. (d) Medical and protective supplies in cities are more abundant than in rural areas, and citizens thus have more opportunities to obtain relevant protective supplies and take protective action.

In mastering the basic knowledge on COVID-19 prevention and control, the ultimate goal is application in practice, i.e., to be able to properly take protective measures, control the roots of infection, cut off the transmission route, and protect vulnerable groups. Multiple linear regression analysis results showed that knowledge, attitude, occupation, education level, and place of residence were the main factors affecting the public’s protective behaviour. According to the KAP model, knowledge is the basis and attitude is the driving force of behaviour change [24]. Therefore, improving people’s knowledge and fostering positive attitudes towards epidemic prevention are indispensable for improving protective behaviour to fight against the COVID-19 pandemic. Moreover, this result indicates that it is necessary to take targeted measures to improve people’s protective behaviour effectively based on their profession, education level and area of residence, especially for non-medical staff, people with low levels of education, and township villagers. These results can be used by health policymakers to develop more targeted policies to fight against the COVID-19 pandemic.

This study has several limitations. First, while we conducted this non-random sampling online survey involving 30 provinces or municipalities by convenience sampling, nearly 80% of the respondents came from Guizhou Province, and the composition of the samples was uneven, mainly including females, medical staff (who have a high education level), city residents and young people. Therefore, the generalizability of the research results has certain limitations. In addition, this study was based on self-reported information about knowledge, attitudes and practices with respect to COVID-19. It is possible that participants looked up the answers before answering some of the questions, which may have exaggerated the accuracy rate of COVID-19 knowledge. To alleviate this bias, we made the questionnaire anonymous and emphasized anonymity during the survey, and we stressed the importance of answering questions honestly before completing the questionnaire. Last, cross-sectional studies are unable to provide evidence on causality. Therefore, the mechanisms for improving COVID-19 protection practices need to be further explored.

## Conclusion

In summary, the public in China has a good grasp of the relevant knowledge on COVID-19, positive attitudes, and high adherence rates with respect to protective behaviours. Practices are affected by knowledge, attitudes, occupation, education, and place of residence.

## Supplementary Information

**Additional file 1.**

## Data Availability

The datasets used and/or analysed during the current study are available from the corresponding author upon reasonable request.

## Abbreviations

COVID-19: Coronavirus disease
KAP: Knowledge, attitudes, and practices
WHO: World Health Organization
CVI: Content validity index
SD: Standard deviation
