# Supplementary material for: Knowledge, attitudes and practices of the Chinese public with respect to coronavirus disease (COVID-19): an online cross-sectional survey
Source: BMC Public Health. 2020 Nov 30;20:1816. doi: 10.1186/s12889-020-09961-2 (PMC7702204; doi:10.1186/s12889-020-09961-2)
Supplement: Supplementary file 1 — Additional file 1. [file 12889_2020_9961_MOESM1_ESM.doc]

**Questionnaire regarding knowledge, attitudes and practices of the Chinese public with respect to coronavirus disease (COVID-19)**

Dear Sir/Madam,
Thank you very much for taking time out of your busy schedule to fill in this questionnaire. The purpose of this survey is to understand the knowledge, attitudes, practices of the Chinese public to COVID-19 infection, so as to provide a scientific basis for the development of corresponding epidemic prevention and control measures in the future. It may take you 3-5 minutes. This survey is conducted anonymously, and the information is absolutely confidential. Please fill it out according to your own opinion. Thank you for your support!

- Would you like to participate in this survey?

□Yes

□No

**Part 1.** **Sociodemographic information**

1. Sex
2. Male
3. Female
4. Age (year):

3. Ethnicity

1. Han
2. Other
3. Marital status
4. unmarried
5. married
6. divorced
7. widowed
8. Education level
9. Middle school and below
10. High school/technical secondary school
11. Junior college
12. Bachelor’s degree
13. Master’s degree and above
14. Occupation
15. Medical staff
16. Worker
17. Famer
18. Self-employed
19. Student
20. Employee of an enterprise or institution
21. Unemployed or retired
22. Other
23. Which province or municipality are you from?
24. Place of residence
25. City
26. Countryside or town

**Part 2. Knowledge level regarding** **COVID-19**

| Items | **True** | **False** | **Don’t know** |
| --- | --- | --- | --- |
| 1. The source of infection is primarily confirmed and asymptomatic patients |  |  |  |
| 1. Transmission mainly occurs through respiratory droplets and close contact |  |  |  |
| 1. The population is generally susceptible to infection |  |  |  |
| 1. It is highly infectious and spreads quickly |  |  |  |
| 1. Washing hands frequently, wearing masks and other measures can effectively prevent infection |  |  |  |
| 1. Family gatherings may spread infection |  |  |  |
| 1. The incubation time of the disease is 1-14 days, typically 3-7 days |  |  |  |
| 1. Most people infected present with fever, fatigue, and dry cough as the main symptoms |  |  |  |
| 1. Most patients have a good prognosis, while a few are in critical condition |  |  |  |
| 1. A suspected case can only be ruled out after two consecutive negative tests of respiratory pathogenic nucleic acid (at least one day apart) |  |  |  |
| 1. Suspected and confirmed patients should be isolated and treated in designated hospitals with effective isolation and protective conditions |  |  |  |
| 1. If you develop a fever during the outbreak, you can take your own medicine |  |  |  |
| 1. Medical alcohol at a concentration of 75% can effectively inactivate the virus |  |  |  |

**Part 3. Attitude towards COVID-19**

| Items | Strongly agree | Agree | Not sure | Disagree | Strongly disgree |
| --- | --- | --- | --- | --- | --- |
| 1. I pay close attention to the development of the epidemic situation |  |  |  |  |  |
| 1. I think I am playing an important role in controlling the epidemic |  |  |  |  |  |
| 1. I fear infection for myself and my family |  |  |  |  |  |
| 1. It is believed that the outbreak will soon be contained |  |  |  |  |  |
| 1. I am willing to cooperate with the relevant departments to take prevention and control measures |  |  |  |  |  |

**Part 4. COVID-19 prevention practices**

| Items | Always | Often | Sometimes | Never |
| --- | --- | --- | --- | --- |
| 1. After the outbreak, stay at home to prevent infection |  |  |  |  |
| 1. Wear a mask when going out |  |  |  |  |
| 1. Wash hands |  |  |  |  |
| 1. Seek medical advice when symptoms such as fever and cough appear |  |  |  |  |
| 1. Monitor body temperature |  |  |  |  |
| 1. Open windows to keep the air fresh |  |  |  |  |
| 1. Rest properly and don't stay up late |  |  |  |  |
| 1. Appropriate exercise |  |  |  |  |
| 1. Home environment disinfection |  |  |  |  |
| 1. Reduce time in airtight, airless environments |  |  |  |  |
| 1. Reduce visits to crowded places |  |  |  |  |
| 1. Avoid direct contact with public facilities that may be infected, such as elevator buttons and stair railings |  |  |  |  |
| 1. Active quarantine after contact with high-risk groups |  |  |  |  |
| 1. Cover mouth and nose when coughing or sneezing |  |  |  |  |
| 1. Keep warm and avoid catching cold |  |  |  |  |
